# Tuning the Pore Geometry of Ordered Mesoporous Carbons for Enhanced Adsorption of Bisphenol-A

**DOI:** 10.3390/ma8041652

**Published:** 2015-04-10

**Authors:** Wannes Libbrecht, Koen Vandaele, Klaartje De Buysser, An Verberckmoes, Joris W. Thybaut, Hilde Poelman, Jeriffa De Clercq, Pascal Van Der Voort

**Affiliations:** 1 Industrial Catalysis and Adsorption Technology (INCAT), Department of Industrial Technology and Construction, Faculty of Engineering and Architecture, Ghent University, Valentin Vaerwyckweg 1, Ghent 9000, Belgium; E-Mails: Wannes.Libbrecht@ugent.be (W.L.); An.Verberckmoes@ugent.be (A.V.); 2 Laboratory for Chemical Technology, Faculty of Engineering and Architecture, Ghent University, Technologiepark 914, Ghent 9052, Belgium; E-Mails: Joris.Thybaut@ugent.be (J.W.T.); Hilde.Poelman@ugent.be (H.P.); 3 Center for Ordered Materials, Organometallics and Catalysis (COMOC), Department of Inorganic and Physical Chemistry, Ghent University, Krijgslaan 281-S3, Ghent 9000, Belgium; E-Mails: Koen.Vandaele@ugent.be (K.V.); Pascal.Vandervoort@ugent.be (P.V.D.V.); 4 Sol–gel Centre for Research on Inorganic Powders and Thin films Synthesis (SCRiPTS), Department of Inorganic and Physical Chemistry, Ghent University, Krijgslaan 281-S3, Ghent 9000, Belgium; E-Mail: Klaartje.Debuysser@ugent.be

**Keywords:** ordered mesoporous carbons, hard template, soft template, adsorption, BPA

## Abstract

Mesoporous carbons were synthesized via both soft and hard template methods and compared to a commercial powder activated carbon (PAC) for the adsorption ability of bisphenol-A (BPA) from an aqueous solution. The commercial PAC had a BET-surface of 1027 m^2^/g with fine pores of 3 nm and less. The hard templated carbon (CMK-3) material had an even higher BET-surface of 1420 m^2^/g with an average pore size of 4 nm. The soft templated carbon (SMC) reached a BET-surface of 476 m^2^/g and a pore size of 7 nm. The maximum observed adsorption capacity (q_max_) of CMK-3 was the highest with 474 mg/g, compared to 290 mg/g for PAC and 154 mg/g for SMC. The difference in adsorption capacities was attributed to the specific surface area and hydrophobicity of the adsorbent. The microporous PAC showed the slowest adsorption, while the ordered mesopores of SMC and CMK-3 enhanced the BPA diffusion into the adsorbent. This difference in adsorption kinetics is caused by the increase in pore diameter. However, CMK-3 with an open geometry consisting of interlinked nanorods allows for even faster intraparticle diffusion.

## 1. Introduction

Porous carbon materials are important in many areas of modern science and technology, with a wide variety of applications, such as catalyst support, electrode material and adsorbents for gas and liquid purification [1]. The popularity of these materials can be attributed to their high specific area, large pore volume, chemical inertness and good mechanical stability [2,3]. Activated carbon (AC) is the most commonly applied carbon material for adsorption due to its low production cost and the possibility for large scale processing. Major disadvantages are the disordered structure, limitation of pore size to micropores (<2 nm) and irregular pore size distribution. The micropores could limit the mass transfer [4], or decrease pore accessibility for larger adsorbates. Further developments in the field of chromatography, Li-based batteries, electrodes and adsorption demand for materials with increased pore sizes and a controlled pore size distribution [5,6]. 

The first hard templated ordered mesoporous that were synthesized, used MCM-48 mesoporous silica molecular sieves as template. The resulting replicated mesoporous carbon CMK-1 exhibited porous structures consisting of two disconnected interwoven three-dimensional pore systems [7]. In a similar manner, a well-defined hexagonally ordered mesoporous carbon denoted CMK-3, was synthesized by using SBA-15 mesoporous silica as template [8]. Next to sucrose solution, as in the original paper [9], also furfuryl alcohol or other carbon precursors such as glucose, xylose, acenaphthene, indene, *etc.* were impregnated in the silica template via the incipient wetness technique [3,10,11]. Therefore, an amount of precursor solution equal to the total pore volume of the template is added. It is believed that the whole solution infiltrated the pores through capillary action. The carbon precursor was typically polymerized by means of a sulfuric acid catalyst and subsequently carbonized under inert conditions. The silica template can be removed either under basic conditions or by means of HF. Unfortunately, in all of the above-mentioned methods, the incipient wetness technique was unsuccessful to fill the pores uniformly due to the difficulty to homogenize a powder. Also pore blocking occurs upon subsequent impregnations of a precursor solution via the wet impregnation method. In order to obtain a high surface area, it is important that no carbon precursor is deposited outside the template because this would lead to external carbon upon calcination.

Soft templated synthesis of ordered mesoporous materials by self-assembly of tri-block copolymer surfactants and carbon precursor was pioneered by Dai *et al.*, Nishiyama *et al.* and Zhao *et al.* [12,13,14,15]. Phenol or resorcinol is polymerized with formaldehyde around the micelles of e.g. pluronic F127 or P123. These amphiphilic surfactants create micelles that adopt tailored symmetries and interact with the carbon precursor. Via an acid or base catalyzed mechanism the precursor reacts into a phenolic resin [2]. The mesostructure formation is aided by the evaporation induced self-assembly (EISA) at acid or neutral pH conditions or by the self-assembly under weakly basic conditions in a hydrothermal synthesis procedure [16]. An increased stability is obtained due to the continuous framework with rigid walls [17]. Variation of morphology is possible by applying different reaction parameters or synthesis mechanisms [1]. Unlike the hard template method, a well-defined symmetry is difficult to achieve for the soft template method. However, under very specific reaction conditions, Zhao *et al.* [18] synthesized a range of (FDU-type) materials with well-defined symmetries. 

To investigate the adsorption capabilities of both mesoporous carbons: soft templated (SMC) and hard templated (CMK-3), BPA was chosen as an adsorbate because it is classified as an endocrine disrupting chemical, putting human health and its reproductive system at risk, which makes the efficient removal of BPA a priority [19,20,21,22]. The adsorption of BPA on activated carbon has been studied intensively [23,24,25], and maximum adsorption capacities (q_max_) from 30 mg/g [25] to as high as 430 mg/g are reported [23]. Important differences are the natural source of the AC, varying from wood [25], to coconut shell [24], to charcoal [23], which will affect the specific surface area as well as surface chemistry by altering the amount of acidic oxygen-containing groups. More recently developed advanced carbon materials tested for BPA adsorption were carbon nanotubes [26] and graphene sheets [27] with a q_max_ of, respectively, 70 mg/g and 182 mg/g.

The objective of this study was to understand the effect of different material characteristics, such as specific surface area, hydrophobicity, pore diameter and pore geometry on the adsorption of BPA by comparing both types of mesoporous carbons and commercial activated carbon. A simple method for the synthesis of CMK-3 was developed. In this synthesis toluene is added after furfuryl alcohol impregnation to avoid the deposit of excess furfuryl alcohol onto the external surface of the silica template. This resulted in a high surface area CMK-3 with well-ordered symmetry. Also, a quick and easy acid-catalyzed EISA synthesis for soft template mesoporous carbon is provided [28]. The synthesis can be performed at room temperature without any pH adjustments or purification steps and results in a well-ordered 2D hexagonal mesoporous material.

## 2. Results and Discussion

### 2.1. Characterization of the Adsorbents

**Figure 1:**
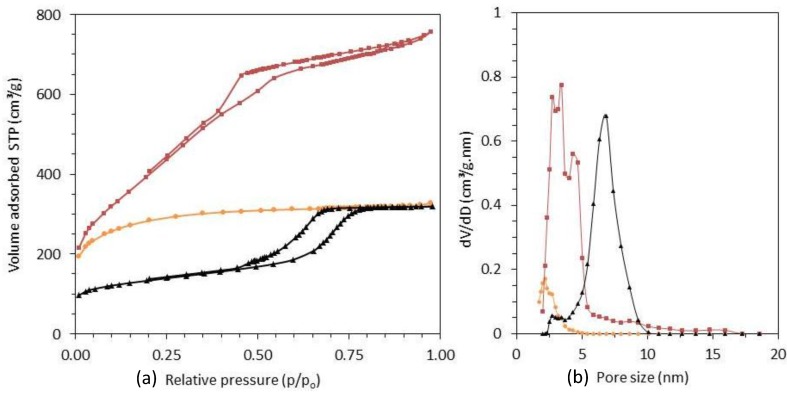
Nitrogen sorption isotherms (**a**) and their corresponding pore size distributions obtained by density functional theory (DFT) method (**b**) of CMK-3 (■), PAC (●) and SMC (▲).

The N_2_-isotherms of the three adsorbents are shown in Figure 1. The BET surface areas, pore volumes, and density functional theory (DFT) pore diameters are listed in Table 1. The PAC surface area and pore volume are 1027 m^2^/g and 0.50 cm^3^/g, respectively, containing mainly micropores, confirmed by the type I isotherm, with a pore diameter below 2.5 nm. The CMK-3 material has a very high surface area (1420 m^2^/g) and pore volume (1.14 cm^3^/g) with a pore diameter of 4 nm. The soft templated mesoporous carbon (SMC) has a surface area of 476 m^2^/g, pore volume of 0.49 cm^3^/g and an average pore diameter of 7.2 nm. Both mesoporous materials exhibit a type IV isotherm with a type H1 hysteresis indicating uniform cylindrical pores. The capillary condensation of CMK-3 occurs at a lower p/p_0_ than SMC indicating a smaller pore size as seen in Figure 1.

**Table 1:** Structural properties of the materials.

Material	Specific Surface Area (m^2^/g)	Pore Volume (cm^3^/g)	Pore diameter D (nm)	Unit cell parameter ^a^ a_0_ (nm)	Wall thickness ^a^ (nm)
PAC	1027	0.50	<3	-	-
CMK-3	1420	1.14	4.0	9.6	5.6
SMC	476	0.49	7.0	11.6	4.6

Note: ^a^ Based on the hexagonal porous structure, the unit cell sizes were calculated by using the formula a = 2d(100)/√3 and pore wall thicknesses were calculated from the formula of h = a − D, with a and D the unit cell parameter and pore diameter, respectively.

**Figure 2:**
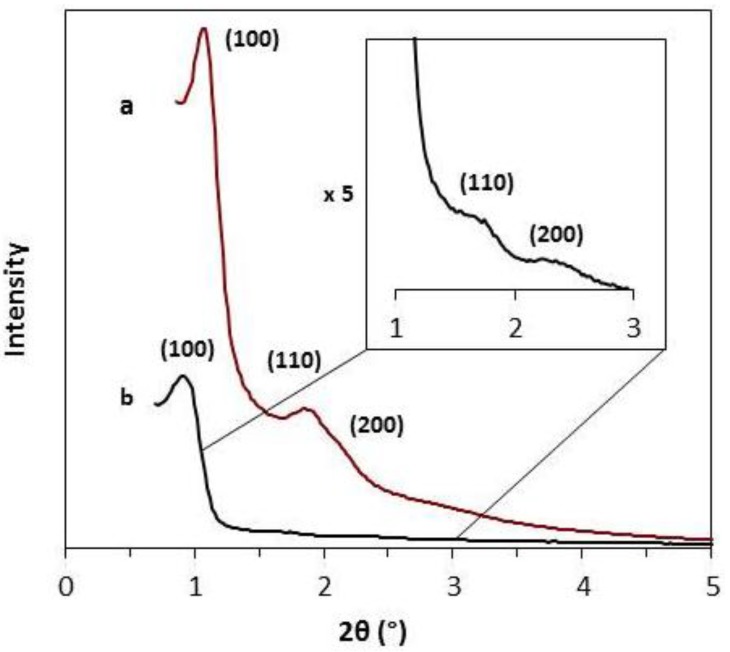
Powder X-ray diffraction patterns of CMK-3 (a) and SMC (b).

**Figure 3:**
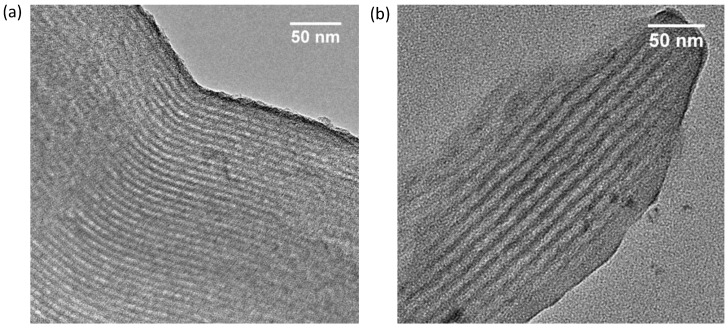
Transmission electron microscope (TEM) images of CMK-3 (**a**) and SMC (**b**).

Powder X-ray diffraction patterns of the synthesized mesoporous carbons are shown in Figure 2. The SMC shows a well resolved (100) reflection at 2θ = 0.88° and high order reflection (110) and (200) of the highly ordered 2D hexagonal symmetry. The CMK-3 material also shows a (100) reflection at 2θ = 1.06° with higher order reflections indicating the 2D hexagonal symmetry. The cell parameter (a_0_) of SMC and CMK-3 were calculated from d(100), d(110) and d(200) and were 11.6 nm and 9.6 nm, respectively. The successful synthesis of highly ordered hard and soft template mesoporous carbons was validated by the TEM images of Figure 3. Both pore size diameters and structures are in agreement with nitrogen sorption isotherms and XRD patterns.

The elemental analysis (Table 2) shows a 93 wt % carbon for both mesoporous carbons. These materials consist mainly of pure carbon walls with a small amount of oxygen containing functional groups. The PAC on the other hand has about 86 wt % carbon and a higher amount of oxygen (Table 2), which is due to the steam activation process during production. The increasing carbon or decreasing oxygen amount indicates a more hydrophobic surface of the mesoporous carbons [23,29]. Because the interactions between BPA and the carbon material are mainly π-π interactions, BPA is expected to adsorb more readily on hydrophobic adsorbents [27,28] 

**Table 2:** Elemental composition of the three carbon adsorbents.

Material	Elemental analysis [wt %]			
	C	H	N	O
PAC	85.90	0.45	0.29	13.36
CMK-3	93.33	0.68	0.07	5.92
SMC	93.93	0.50	0.08	5.49

### 2.2. Adsorption Isotherms

**Figure 4:**
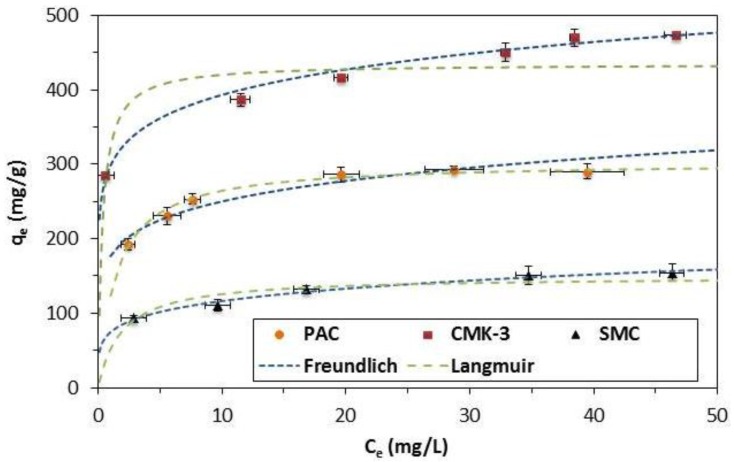
Adsorption isotherms of 10 mg of CMK-3 (■), PAC (●) and SMC (▲) in 100 mL of bisphenol-A (BPA)-solution, experimental data were fitted with the Langmuir and Freundlich model, the error bars represent the standard deviation of the triplicate measurements.

Figure 4 shows the adsorption isotherms of CMK-3, SMC and PAC. CMK-3 has the highest adsorption capacity, followed by PAC and SMC. The experimental data were fitted with Langmuir and Freundlich isotherms (Table 3). The isotherms of the mesoporous materials are better described by the Freundlich model (Equation (3)), while the experimental PAC-data are better described by the Langmuir model (Equation (2)). This implies multilayer adsorption could occur within the wide pores of the mesoporous materials, while for PAC, mainly consisting of micropores, adsorption will be limited to a monolayer. The observed maximum adsorption capacities are 474 mg/g, 290 mg/g, and 154 mg/g for, respectively, CMK-3, PAC and SMC. To the best of our knowledge the adsorption capacity of 474 mg/g for CMK-3 is the highest reported. BPA adsorption from water on a carbon adsorbent is mainly influenced by specific surface area and hydrophobicity of the surface due to π-π interactions between BPA and the adsorbate [23,27,28]. As the specific surface area increases from SMC, over PAC to CMK-3 (Table 1), the adsorbed BPA amount increases. To compare the materials without the effect of specific surface area, the observed maximum adsorption capacities are converted to the number of adsorbed molecules per nm^2^ (Table 3). The more hydrophobic mesoporous carbons, CMK-3 and SMC, with about 94 wt % carbon, have a higher amount of molecules adsorbed per nm^2^: 0.88 molecules/nm^2^ and 0.86 molecules/nm^2^ compared to the less hydrophobic PAC (0.75 molecules per nm^2^). 

**Table 3:** The observed maximum adsorption capacity in mg/g and converted to molecules per nm^2^, parameters describing the adsorption isotherms of BPA on PAC, CMK-3 and SMC modeled by Langmuir and Freundlich.

Material	Experimental		Langmuir			Freundlich		
	q_max_ (mg/g)	Adsorbed BPA (molecules/nm^2^)	q_max_ (mg/g)	K_L_ (L/mg)	R^2^	1/n	K_F_ (mg/g (L/mg)^1/n^)	R^2^
PAC	290	0.75	307	0.64	0.98	0.14	181	0.93
CMK-3	474	0.88	447	2.81	0.82	0.12	296	0.99
SMC	154	0.86	156	0.40	0.87	0.19	74	0.98

### 2.3. Adsorption Kinetics

**Figure 5:**
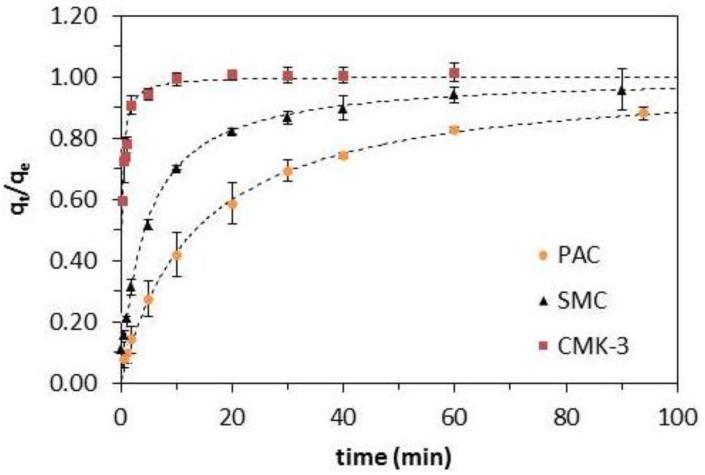
Adsorption kinetics of 100 mg of CMK-3 (■), PAC (●) and SMC (▲) in 1 L of 60 mg/L BPA-solution, particle size is between 62 and 88 µm, the error bars represent the standard deviation of the triplicate measurements, the dashed lines represent the pseudo-second-order kinetic model descriptions.

The adsorption kinetics of BPA on PAC, CMK-3 and SMC for the smallest particle size (62–88 µm) are shown in Figure 5, where the adsorption capacity q_t_, normalized to the equilibrium adsorption capacity q_e_ is given as function of time. This allows comparison of the three materials with different equilibrium adsorption capacities (q_e_) and therefore a different driving force for BPA removal. It is clear that adsorption on CMK-3 is the fastest, reaching equilibrium after 20 min. SMC reaches equilibrium at about 60 min, while PAC has not yet reached equilibrium after 90 min. It is noteworthy that SMC adsorbs faster than PAC, although SMC has a lower equilibrium adsorption capacity than PAC: 154 mg/g *versus* 290 mg/g. The experimental data were fitted with the pseudo-first-order, pseudo-second-order and Weber-Morris kinetic model (Table 4). The kinetics for all three materials were best described by the pseudo-second-order kinetic model. The adsorption kinetics of organic pollutants on carbon materials has often been described with pseudo-second-order kinetics [4,23,24,27]. Based on the Weber-Morris kinetic model, a plot of q_t_ *versus* t^1/2^ should be linear if intraparticle diffusion was involved in the adsorption process. Moreover, if the straight line passes through the origin, intraparticle diffusion is the rate-determining step. Only the PAC data showed a straight line, with an extrapolation passing almost through the origin. This suggests that intraparticle diffusion contributes to the adsorption kinetics of PAC.

**Table 4:** Parameters of the pseudo-first-order, pseudo-second-order and Weber-Morris kinetic models for adsorption of BPA on PAC, CMK-3 and SMC.

Material		Pseudo-first-order model			Pseudo-second-order model				Weber-Morris		
	q_e_ (exp) (mg/g)	q_e_ (calc) (mg/g)	k_1_ (1/min)	R^2^	q_e_ (calc) (mg/g)	k_2_ (g/(mg∙min)	t_1/2_ (min)	R^2^	k_d_ (min∙g/(mg))	C (mg/g)	R^2^
PAC	277	264	0.0657	0.98	315	0.00024	13.4	0.99	31.2	13.2	0.94
CMK-3	295	281	2.64	0.77	291	0.01647	0.208	0.96	13.7	215	0.65
SMC	147	137	0.0236	0.97	149	0.00157	4.27	0.99	15.0	29.6	0.86

**Figure 6:**
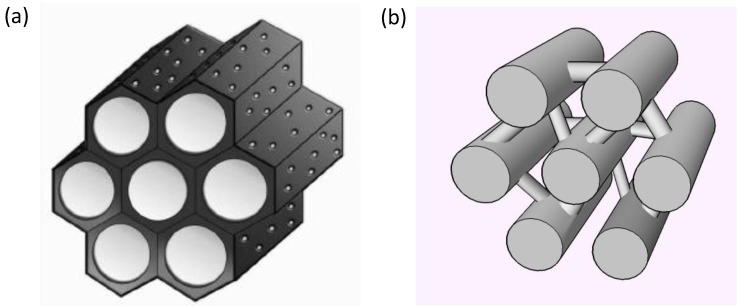
(**a**) Schematic representation of 2D hexagonal mesoporous structure of SMC and (**b**) the interconnected nanorod structure of CMK-3.

Although the BPA-molecule with molecular dimensions of 1.068 nm × 0.587 nm × 0.383 nm [25,27,30,31] is able to enter micropores and PAC has been reported as a successful adsorbent in multiple studies [23,24,32], the observed adsorption kinetics in Figure 5 show that the larger pores of mesoporous carbons increase the efficiency of these materials beyond that of PAC. This is consistent with literature reporting that the use of mesoporous materials as adsorbent has proven to increase adsorption rate and efficiencies [4,29,33]. The BPA molecule diffuses more readily in the larger mesopores and is adsorbed faster. The difference in rate between CMK-3 and SMC can be attributed to the pore geometry. SMC consists of 2D hexagonal arranged long pores, while CMK-3 consists of interconnected nanorods (Figure 6). This open pore system of CMK-3 allows for a faster intraparticle diffusion and removal rate of the adsorbate, as observed in Figure 5.

**Figure 7:**
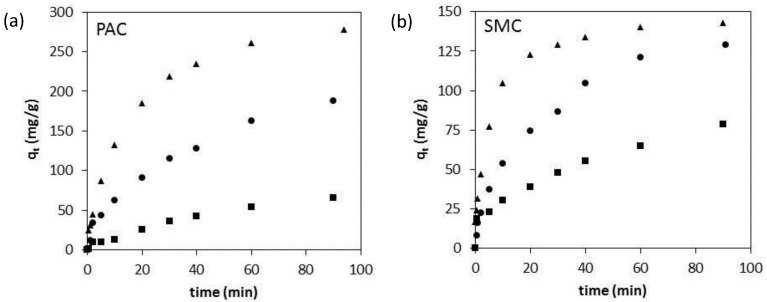
Effect of particle size 62–88 µm (▲), 177–250 µm (●) and 510–700 µm (■), on the adsorption kinetics of 100 mg of PAC (**a**) or SMC (**b**) in 1 L of 60 mg/L BPA-solution.

The effect of particle size on the adsorption kinetics was further investigated for SMC and PAC (Figure 7). With increasing particle size, the removal rate of BPA on PAC is considerably lowered. The irregular structured micropores of the PAC particles clearly indicate that intraparticle diffusion is the rate-controlling step. This was also confirmed by fitting the PAC data of Figure 7 to the Weber-Morris kinetic model (Table 5). The removal rate of BPA on SMC is higher than that of PAC, because the mesopores allow a better diffusion through the material. Remarkably, in the SMC with larger pores and a well-ordered structure, intraparticle diffusion also controls the removal rate (Figure 7). This could be explained by the pore structure: in larger particles, the average distance travelled by diffusion in the long 2D hexagonal pores will be longer. Fitting the SMC data of Figure 7 to the Weber-Morris kinetic model confirmed the contribution of intraparticle diffusion in larger SMC particles (Table 5).

**Table 5:** Parameters of the Weber-Morris kinetic model for adsorption of BPA on PAC and SMC with different particle sizes.

PAC	k_d_ (min∙g/mg)	C	R^2^	SMC	k_d_ (min∙g/mg)	C	R^2^
62–88 µm	31.2	13.2	0.94	62–88 µm	15.0	29.6	0.86
177–250 µm	20.9	−3.93	0.99	177–250 µm	13.5	6.10	0.97
510–700 µm	7.62	−6.68	0.98	510–700 µm	8.33	2.60	0.99

## 3. Experimental Section

### 3.1. Synthesis of Ordered Mesoporous Carbons

A commercial activated carbon was supplied by Desotec (Roeselare, Belgium). For the hard templated mesoporous carbon CMK-3 synthesis, 2.5 g of the template SBA-15 was impregnated with 10 mL solution of 25 v % furfuryl alcohol (≥98% purity, Sigma-Aldrich, Steinheim, Germany) in toluene (≥99.5% purity, Sigma-Aldrich). To catalyze the reaction, 200 µL of 0.1 mol/L H_2_SO_4_ (Sigma-Aldrich) is added. The solution was refluxed for 36 h at 90 °C while stirring vigorously. Next, the impregnated silica is filtered and washed 3 times with toluene and dried at 150 °C for 5 h in a Nabertherm muffle oven. The composite material is carbonized at 1100 °C for 1 h in a Carbolite tubular furnace under Ar—5% H_2_ atmosphere with a heating rate of 2 °C/min. The silica is leached out by stirring with a 10% HF solution (Sigma-Aldrich) for a minimum of 6 h. Less than 3 wt % SiO_2_ was detected in the CMK-3 by X-ray fluorescence (Rigaku NEX CG, Tokyo, Japan). 

The soft templated mesoporous carbon synthesis was based on Wang *et al.* [34]. Resorcinol (2.2 g; Sigma-Aldrich) and F127 (2.2 g; Sigma-Aldrich) were mixed with 9 mL ethanol (≥99.8% purity VWR) and 9 mL HCl (3 mol/L) and stirring for 15 min at room temperature. Then, formaldehyde (2.6 g) (Formalin, 37 wt % formaldehyde in water; Sigma-Aldrich) was added and the solution was stirred again for 15 min. The solution was subsequently poured onto a glass plate to evaporate the ethanol at room temperature for 6 h. Next, the film was scraped from the glass plate and put into a petri-dish in a Nabertherm muffle oven where the material was cured at 60 °C for 12 h. The cured resin was calcined and carbonized under N_2_-flow in a Thermolite tubular furnace with a heating rate of 1 °C/min. In the first heating step to 350 °C for 2 h, the surfactant was removed. The second heating step to 800 °C for 3 h carbonized the resorcinol/formaldehyde resin. 

### 3.2. Characterization

Powder X-ray diffraction (PXRD) patterns of the adsorbents were collected on a Thermo Scientific ARL X’Tra diffractometer, operated at 40 kV, 40 mA using Cu Kα radiation. The PXRD patterns were measured from 0.6° to 5° with a step size of 0.02°. Nitrogen gas sorption experiments were conducted at −196 °C using a Micromeritics Tristar 3000. Samples were vacuum-dried at 120 °C overnight prior to analysis. The pore size distribution (PSD) was calculated with the DFT method using a carbon kernel (Microactive software, Micromeritics). Total pore volume V_tot_ was calculated as the amount of nitrogen adsorbed at a relative pressure of 0.95. The total surface area S_BET_ was determined using the Brunauer–Emmett–Teller (BET) method. Elemental analysis was performed with a CHNS-O analyzer Thermo Scientific Flash 2000, with a TCD detector, using the Eager Experience software. Images of the ordered mesopores were obtained by a TEM (Jeol JEM Cs-corrected 2200FS, Peabody, MA, USA, operating at 200 kV).

### 3.3. Adsorption Tests

Kinetic experiments were performed by adding 100 mg of adsorbent with a particle size of 62 to 88 µm to 1 L of 30 mg/L BPA (>99% purity; Sigma-Aldrich) solution. These aqueous BPA solutions were stirred at room temperature at 600 rpm. 5 mL samples were taken at different time intervals up to 100 min, filtered through a 0.45 µm PET syringe filter and the BPA concentrations were analyzed with a Thermo scientific Evolution 60 UV/VIS spectrophotometer at a wavelength of 275 nm. In order to ascertain that the obtained data are free from external diffusion limitations across the solid-liquid interface, preliminary kinetic experiments at different agitation speeds (300, 600 and 900 rpm) confirmed that external mass transfer resistance is negligible from an agitation speed of 600 rpm for all three adsorbents (results not shown) [35].

The internal mass-transfer effect was studied by using different adsorbent particle sizes for PAC and SMC. Both materials were crushed and sieved in three different particle sizes (62–88 µm, 177–250 µm and 510–700 µm) with a Mini-sieve micro sieve set (Sigma-Aldrich). Because CMK-3 is a very fine powder, only the smallest particle size fraction was available. Pelletizing of CMK-3 gave larger particle sizes, however upon mixing in solution the particles disintegrated back to the very fine powder. 

Isotherm experiments were performed by adding 10 mg of adsorbent to 100 mL of aqueous BPA solution with different BPA concentrations (ranging from 5 mg/L to 70 mg/L). Subsequently, the solutions were placed in a thermostatic shaking device (Infors HT, multitron standard) at 25 °C for 24 h. Next, the solutions were filtered through a 0.45 µm PET syringe filter and analyzed for its BPA concentrations.

Both kinetic and isotherm experiments were performed in triplicate.

The adsorption capacity at time t was calculated according to Equation (1):

(1) $$q_{t}=\frac{(C_{0}-C_{t})V}{m}$$

with *q_t_* (mg/g) the adsorption capacity at time *t*; *C_0_* (mg/L) and *C_t_* (mg/L) the initial concentration and concentration at time *t*; *m* (g) the adsorbent mass and *V* (L) the solution volume. 

The isotherm data were fitted to the well-known Langmuir (Equation (2)) and Freundlich (Equation (3)) isotherms:

(2) $$q_{e}=\frac{q_{\max}K_{L}C_{e}}{1+K_{L}C_{e}}$$

(3) $$q_{e}=K_{F}C_{e}^{1/n}$$

with q_max_ (mg/g) the maximum adsorption capacity; *C_e_* (mg/L) the equilibrium concentration; K_L_ (L/mg) the Langmuir constant; K_F_ (mg/g∙(L/mg)^1/n^) and n (-) the Freundlich constants. 

The kinetic data were fitted to the pseudo-first-order (Equation (4)), the pseudo-second-order (Equation (4)), and the Weber-Morris kinetic model (Equation (6)):

(4) $$q_{t}=q_{e}(1-e^{-k_{1}t})$$

(5) $$q_{t}=\frac{q_{e}t}{\frac{1}{k_{2}q_{e}}+t}$$

(6) $$q_{t}=k_{d}t^{1/2}+C$$

with k_1_ (1/min) the pseudo-first-order rate constant; k_2_ (g/(mg∙min)) the pseudo-second-order rate constant; k_d_ (min∙g/mg) the intraparticle diffusion rate constant and C (mg/g) the intercept.

## 4. Conclusions

Commercial activated carbon and synthesized hard and soft templated mesoporous carbons were fully characterized and tested for their BPA adsorption capacity and kinetics. Both mesoporous materials showed a well-ordered 2D hexagonal structure, SMC consists of cylindrical pores with a size of 7.0 nm while CMK-3 has an interconnected nanorod structure with an average pore size of 4.0 nm. The observed maximum adsorption capacity increased with the specific surface area of the material, CMK-3 showed a very high adsorption capacity of 474 mg/g, and outperforms PAC with an adsorption capacity of 290 mg/g. When calculating the number of molecules adsorbed per nm^2^, both mesoporous materials, *i.e.*, SMC and CMK-3, performed better than PAC. This difference was attributed to the more hydrophobic nature of SMC and CMK-3 compared to PAC. The adsorption kinetics showed a clear advantage of mesoporous materials over PAC. The BPA removal rate of CMK-3 was fastest followed by SMC and PAC. For both SMC and PAC the increase in particle size resulted in a slower BPA removal, which shows that the adsorption is controlled by intraparticle diffusion, although this effect was more pronounced for PAC than SMC. In conclusion, mesoporous carbons are promising adsorbents for the removal of BPA, especially CMK-3 with its interconnected nanorod structure and high specific surface area resulting in the highest removal rate as well as adsorption capacity. 
